# Partial Ceramic Veneers as a Conservative Restorative Strategy: A Narrative Review with Case Report

**DOI:** 10.3390/dj14030186

**Published:** 2026-03-23

**Authors:** Jose Villalobos-Tinoco, Carlos A. Jurado, Mark Adam Antal, Silvia Rojas-Rueda, Hamid Nurrohman

**Affiliations:** 1 Department of Restorative Dentistry, Center for Dental Studies (CEO), Queretaro 76050, Mexico; 2 Independent Researcher, Culiacan 80030, Mexico; 3 Division of Operative Dent, Department of General Dentistry, College of Dentistry, University of Tennessee Health Science Center, Memphis, TN 38104, USA; 4 School of Dental Medicine, Ponce Health Sciences University, Ponce 00732, Puerto Rico; 5 Department of Operative and Esthetic Dentistry, Faculty of Dentistry, University of Szeged, 6720 Szeged, Hungary; 6 Division of Dental Biomaterials, Department of Clinical and Community Sciences, School of Dentistry, University of Alabama at Birmingham, Birmingham, AL 35233, USA; 7 Department of Restorative Dentistry & Prosthodontics, The University of Texas School of Dentistry, Houston, TX 77054, USA

**Keywords:** ceramic veneers, partial veneers, dental adhesion, esthetic dentistry

## Abstract

**Background:** Partial ceramic veneers in the esthetic zone are a novel, conservative alternative to traditional veneer preparations intended to preserve maximum tooth structure. This narrative review summarizes the available clinical case reports on partial ceramic veneers and includes a case illustration demonstrating a step-by-step approach to closing a space between the maxillary left lateral incisor and canine. **Methods:** The review synthesizes the limited case-report evidence, focusing on patient selection, treatment planning, and clinical execution. The case illustration details each step, including a diagnostic digital wax-up to preview the proposed outcome and a minimally invasive preparation limited to rounding sharp areas and optimizing the path of insertion. **Results:** Published reports emphasize that careful case selection and a well-executed plan are essential. In the case illustration, hand-crafted partial veneers achieved a natural appearance, with a high esthetic outcome confirmed using the White Esthetic Score (WES) system. **Conclusions:** Although evidence remains limited, partial ceramic veneers can be predictable in appropriately selected cases. More long-term clinical data are needed, and the case illustration may help guide early-career clinicians. The case illustration is limited in that it does not provide quantifiable outcomes like in vitro studies; however, qualitatively, it fulfilled the patient’s esthetic and functional demands.

## 1. Introduction

Facial attractiveness has been shown to positively influence a wide range of social and professional outcomes, shaping how individuals are perceived and treated in everyday interactions. Appearance-related impressions can affect the ease of forming friendships, social acceptance, and evaluations in professional settings such as hiring or advancement [1,2]. Because the face is central to communication, even subtle differences may influence judgments of approachability, trustworthiness, and competence, affecting opportunities in both social and occupational contexts [1,2]. Among the contributors to facial attractiveness, the smile is central to dentofacial harmony, integrating dental form, gingival display, and lip posture into a highly visible feature. An attractive smile has been associated with improved self-esteem and confidence, particularly among young adults [3].

In recent years, social media has amplified the perceived importance of an attractive smile [4,5]. Visual platforms promote rapidly evolving beauty standards and frequent comparison, increasing awareness of dental esthetics [4,5]. Consequently, patients are increasingly attentive to tooth shade, symmetry, incisal edge position, and the smile arc, and often seek conservative, predictable treatments that deliver natural-looking results.

Ceramic veneers are a widely accepted option for addressing esthetic concerns while preserving tooth structure, particularly for improving color, shape, and harmony. They are commonly used for discoloration, mild wear, localized defects, non-ideal contours (e.g., peg laterals or uneven incisal edges), and diastemas [6,7,8,9]. Their optical properties (translucency and light diffusion) can closely mimic enamel and support highly natural outcomes when material selection and layering are appropriate [6,7,8,9]. Veneers are generally more conservative than full-coverage crowns, typically requiring removal of 3% to 30% of coronal tooth structure, compared with approximately 63% to 72% for crowns [10]. Enamel preservation improves bonding predictability and reduces biologic risk, including unnecessary dentin exposure or pulpal irritation. Long-term data show that ceramic veneers can be durable when case selection, occlusion, and bonding protocols are properly managed, with survival rates reported as high as 95% at 10 years [11]. However, success is technique-sensitive and depends on factors such as adhesive quality, functional loading (including parafunction), material thickness/design, and careful finishing/polishing to support periodontal compatibility and a harmonious emergence profile. Partial veneer preparations are a newer, highly conservative approach, often involving minimal or no reduction and no definitive finish line. They restore only the compromised or esthetically deficient area—such as a localized proximal deficiency, minor contour discrepancy, limited fracture, or small space—while preserving healthy structure [12,13]. Although conceptually additive and enamel-preserving, they increase clinical and laboratory complexity because the ceramic–tooth transition must be imperceptible in shade, translucency, texture, and smoothness. Without a defined finish line, margin identification and design are more demanding, and a visible junction may occur if ceramic thickness, characterization, or cement shade is not carefully controlled. Cementation is particularly critical, as cement thickness, polymerization, and finishing directly affect esthetics and surface continuity. Despite growing interest, the literature remains limited and lacks well-defined protocols for patient selection, preparation (when indicated), and bonding/cementation sequences [12,13]. Therefore, this manuscript is justified by the limited clinical guidance currently available for partial ceramic veneers in the esthetic zone. It presents a case illustration in which two partial ceramic veneers were used to close a diastema between the maxillary left canine and lateral incisor, demonstrating a conservative approach to optimize esthetics while preserving tooth structure. In addition, it includes a narrative review of the existing clinical reports on partial veneers in the esthetic zone, summarizing their methodologies and key conclusions. It should be noted that this review represents a narrative synthesis of selected clinical reports and does not aim to provide a fully systematic or exhaustive evaluation of the available evidence.

## 2. Materials and Methods

### 2.1. Literature Review

A literature search was conducted to identify case reports published between January 2010 and December 2025 describing partial ceramic veneers in the smile (esthetic) zone. The search was performed in PubMed, Scopus, Web of Science, and Google Scholar using the following terms: “Partial Ceramic Veneers,” “Partial Labial Veneers,” and “Partial Laminate Veneers.” The initial search yielded 68 articles. Titles and abstracts were screened, and five manuscripts were selected for full-text review. Only case reports that provided a detailed, step-by-step description of the clinical procedure were included.

Several exclusion criteria were applied. Publications prior to January 2010, non-English articles, letters, books, book chapters, and manuscripts without full-text availability were excluded. The inclusion and exclusion criteria are detailed in Table 1. This selection process ensured the inclusion of relevant, high-quality case illustrations, enabling a focused analysis of the clinical steps and outcomes of partial ceramic veneers in the esthetic zone. The selected manuscripts provide insights into clinical outcomes, patient satisfaction, and procedural developments related to this novel, conservative approach.

It is important to mention that a narrative review provides a comprehensive—often interpretive—overview of a topic, typically written by subject-matter experts. It emphasizes synthesis, critique, and thematic interpretation rather than a rigid, fully replicable methodology. Unlike systematic reviews, narrative reviews generally do not follow predefined protocols (e.g., PROSPERO registration), exhaustive search strategies, or formal quality appraisal procedures [14].

Because narrative reviews are non-systematic, they typically do not require PRISMA (Preferred Reporting Items for Systematic Reviews and Meta-Analyses) reporting. In contrast to systematic reviews, they are not expected to document a fully reproducible process (such as exact search terms, databases searched, or explicit inclusion/exclusion criteria presented in a PRISMA flow diagram). Narrative reviews allow authors to summarize what is known while offering informed critique of the broader literature. They can describe the current state of a field and highlight emerging directions, new theories, or alternative interpretations of existing evidence. As a result, narrative reviews are valuable for under-researched topics and for generating fresh insights in well-established areas—especially when the evidence base is broad, complex, or requires nuanced interpretation [15,16].

### 2.2. Methodological Assessment

The selected case illustration was analyzed for methodological characteristics and out-come reporting. The following variables were assessed: use of partial veneers, endodontic treatment involvement, implant involvement, description of standardized adhesive protocols, follow-up reporting, and availability of outcome data. Each variable was categorized as reported (+) or not reported (−). An overall assessment was calculated based on the total number of reported items.

### 2.3. Initial Exam and Treatment Planning

**Figure 1:**
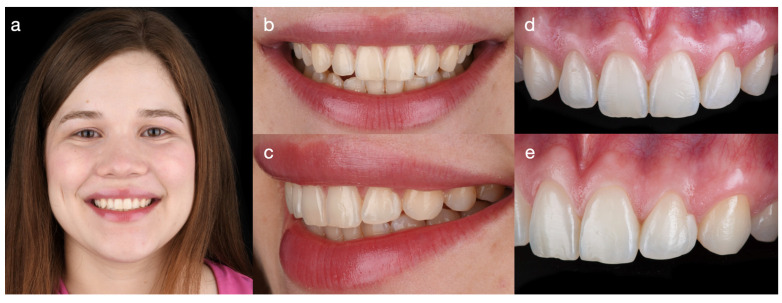
Baseline clinical photographs demonstrate the non-ideal proportions of the resin composite restoration on the maxillary left lateral incisor placed to close the diastema. (**a**) Full-face smile, (**b**) frontal smile view, (**c**) right lateral smile view, (**d**) intraoral frontal view, and (**e**) intraoral left lateral view.

A 30-year-old female patient presented with the chief complaint of disliking a previous resin composite restoration in the esthetic zone. The patient reported having received a direct resin composite on the maxillary left lateral incisor four years earlier to close the space between the lateral incisor and canine. However, the restoration created a non-ideal tooth dimension, and an overhang allowed food to accumulate and made the area difficult to clean (Figure 1).

The patient was offered the option of replacing the resin composite restoration with ceramic veneers. She was informed that the space between the lateral incisor and canine could be divided and restored with two ceramic veneers, providing more ideal proportions for both teeth. The veneers would be designed as partial veneers with no defined finish line to maintain a minimally invasive approach.

Gingivectomy of the maxillary left canine was recommended to improve the gingival architecture, and the patient was also offered tooth whitening prior to veneer placement to enhance the overall shade of the anterior teeth. The patient accepted the treatment plan with the gingivectomy procedure and, finally, the placement of two ceramic partial veneers.

### 2.4. Digital Wax-Up and Gingivectomy Guide

**Figure 2:**
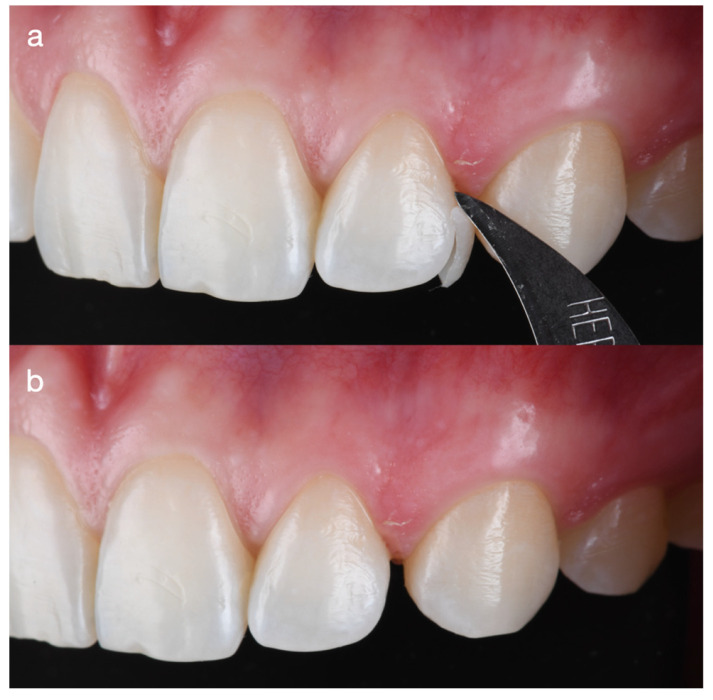
Removal of the overcontoured resin composite on the distal aspect of the maxillary left lateral incisor, which created non-ideal tooth proportions and an unnatural appearance. (**a**) The restoration was removed using a blade; (**b**) following removal, the space between the lateral incisor and the canine is evident.

First, the old composite restoration on the distal surface of the maxillary left lateral incisor was removed (Figure 2).

**Figure 3:**
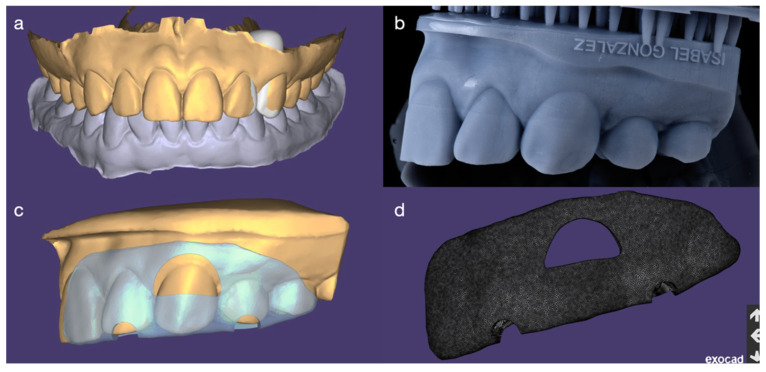
Digital workflow for the diagnostic wax-up and gingivectomy guide design based on an intraoral scan. The guide was fabricated to facilitate gingivectomy and optimize gingival contours around the maxillary left canine prior to definitive restorations. (**a**) Digital wax-up (frontal view); (**b**) printed model with wax-up contours; (**c**) virtual design of the gingivectomy guide on the wax-up model; and (**d**) gingivectomy guide shown alone.

Intra-oral scan was performed (Medit i600, Medit, Seoul, South Korea), and a digital wax-up (3.1 Rijeka, Exocad DentalCAD, Darmstadt, Germany) was performed in order to close the space evenly between the maxillary left lateral incisor and left canine. Also, a gingivectomy guide was designed with a window on the zenith level of the maxillary left canine. Lastly a diagnostic model of the digital wax-up was printed as well as the gingivectomy guide (Figure 3).

Because the teeth were well aligned within the arch and the diagnostic digital wax-up showed that closing the gap between the maxillary left lateral incisor and canine would still maintain natural tooth contours, the patient was informed that partial ceramic veneers could provide natural-looking restorations.

### 2.5. Gingivectomy and Tooth Preparation

**Figure 4:**
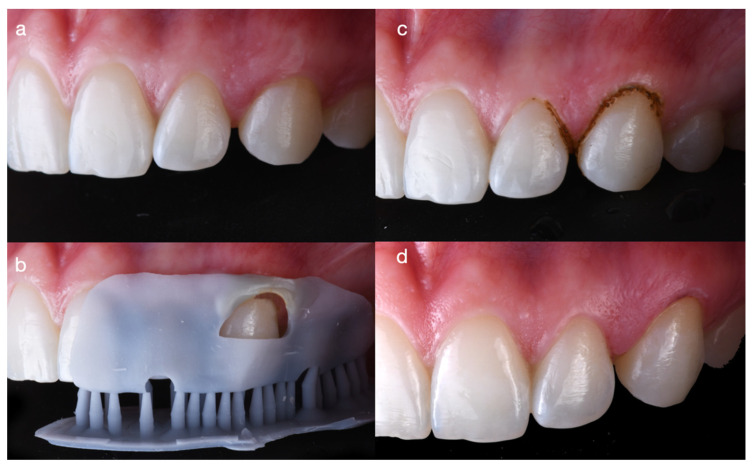
Gingivoplasty performed with a 3D-printed guide to optimize gingival contours and enhance the natural appearance of the teeth. (**a**) Baseline presentation; (**b**) placement of the 3D-printed guide; (**c**) gingivoplasty performed according to the guide; and (**d**) final outcome showing improved, natural-looking soft-tissue contours.

The printed gingivectomy guide was placed intraorally, and gingivectomy was performed with an electrosurgical unit (700SE Electrosurge, Parkell, Edgewood, NY, USA) following the contours of the guide. The outcome significantly improved the gingival architecture, achieving the desired result of positioning the gingival zenith higher than that of the lateral incisor (Figure 4).

**Figure 5:**
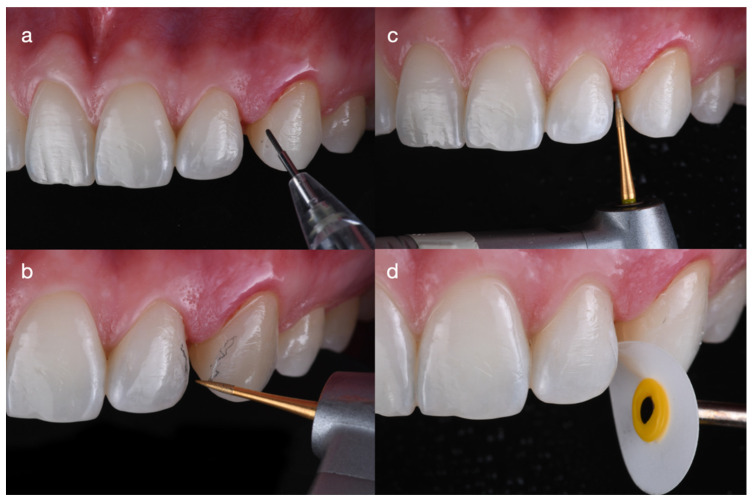
Minimally invasive tooth preparation prior to the final impression for partial ceramic veneers. (**a**) Line angles marked with a pencil; (**b**) facial refinement to eliminate sharp angles that may interfere with veneer seating; (**c**) interproximal finishing to ensure smooth, well-defined margins; and (**d**) polishing of the final preparation.

Minimally invasive tooth preparations were then completed. First, the distal line angle of the maxillary left lateral incisor and the mesial line angle of the left canine were marked with a pencil to guide the preparation (Diatech Inlay & Crown Preparation Kit, Coltene, Altstätten, Switzerland), ensuring an appropriate path of insertion for the veneers. All surface irregularities were removed, and the preparations were polished with polishing disks (OptiDisc, Kerr, Brea, CA, USA) (Figure 5).

**Figure 6:**
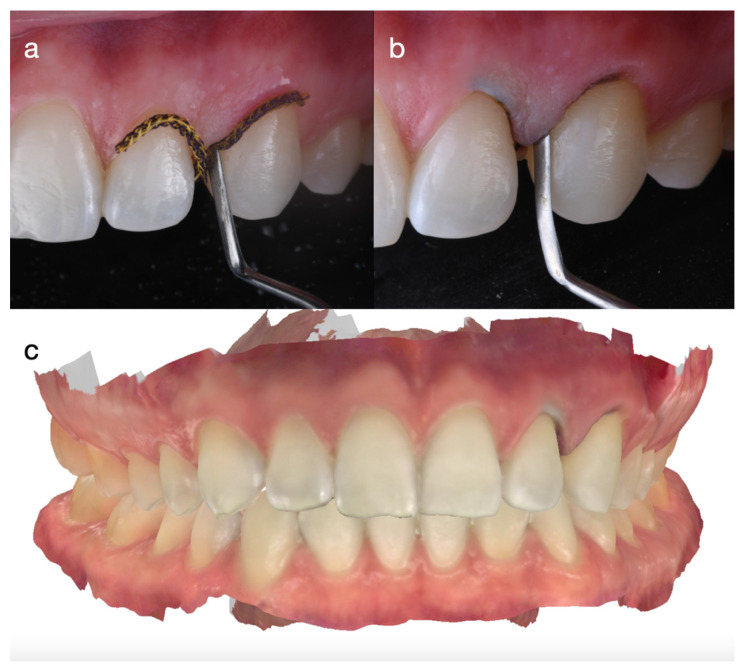
Gingival retraction cord placement to achieve soft-tissue displacement for the final digital impression. (**a**) Initial cord placement; (**b**) cord fully seated within the sulcus; and (**c**) final digital impression used for fabrication of the definitive partial ceramic veneers.

Finally, a retraction cord (Ultrapak, Ultradent, South Jordan, UT, USA) was packed around the maxillary left lateral incisor and left canine, and a final digital impression was taken using the scanner (Figure 6).

### 2.6. Fabrication and Cementation of Restorations

**Figure 7:**
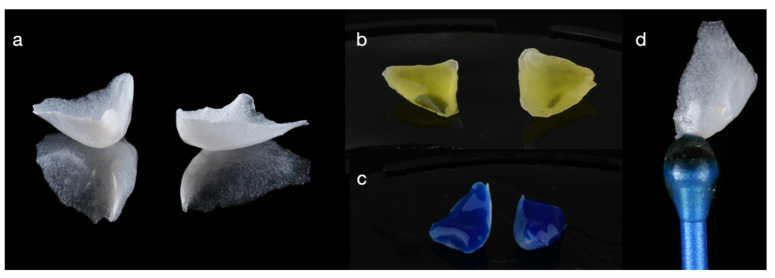
Fabricated partial ceramic veneers without a distinct finish line, unlike conventional full veneers. (**a**) Veneer prior to surface conditioning, (**b**) hydrofluoric acid etching for 20 s, (**c**) phosphoric acid application to remove residual etching byproducts after HF etching, and (**d**) final view of the partial ceramic veneer restoration.

A 3D printed model (Anycubic Resin 3D Printer Mono 4K, Anycubic, Shenzhen, China) was obtained from the final digital impression, and it was duplicated (Type IV Stone, Fujirock, GC, Tokyo, Japan). The partial ceramic veneer restorations were fabricated with press technique out of feldspathic porcelain (Noritake Super Porcelain EX-3, Kuraray Dental, Tokyo, Japan). Then restorations were first treated with hydrofluoric acid (Ceramic Etching Gel, Ivoclar, Schaan, Liechtenstein) for 60 s, and then cleaned with phosphoric acid (Uni-Etch w/BAC, Bisco Dental, Schaumburg, IL, USA) for 60 s, and then placed in ultrasonic bath (5300 Sweep Ultrasonic Cleaner, Quala Dental Products, Nashville, TN, USA) with alcohol for 5 min (Figure 7).

**Figure 8:**
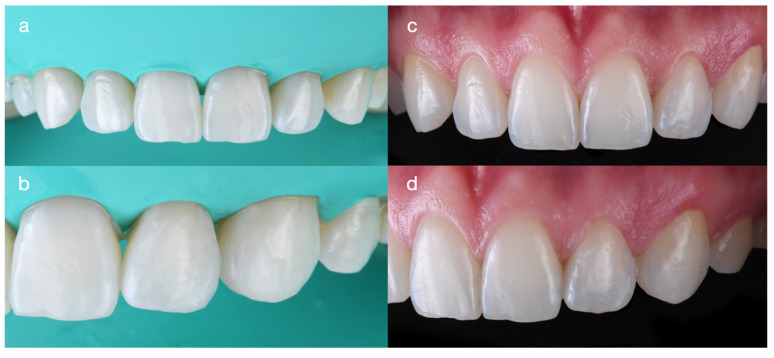
Cementation of partial ceramic veneers under complete rubber dam isolation to minimize contamination and optimize adhesive bonding. (**a**) Rubber dam placement, (**b**) close-up of the restorations immediately after cementation, and (**c**) frontal and (**d**) lateral views following rubber dam removal.

Total isolation was achieved using a dental dam (Nico Tone Dental Dam, MDC Dental, Guadalajara, Mexico) secured with clamps on the maxillary left and right first molars. The tooth surfaces were first cleaned with pumice paste (Pumice Preppies, Whip Mix, Louisville, KY, USA). The restorations were then cemented using resin cement (Variolink Esthetic LC, Ivoclar, Schaan, Liechtenstein), and the excess cement was removed before light curing for 20 s on each surface, including the facial, incisal, and interproximal areas. The cemented restorations were polished, and occlusion was evaluated and adjusted as needed (Figure 8).

### 2.7. Outcome

**Figure 9:**
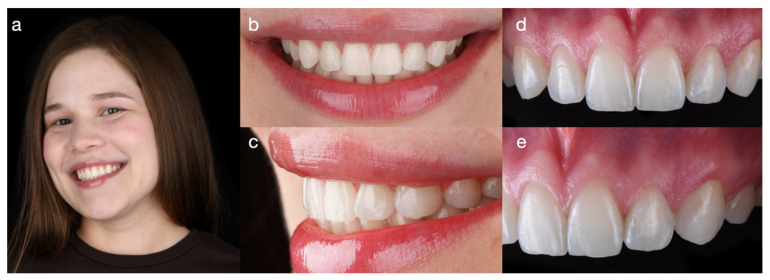
Final outcome following placement of partial ceramic veneers on the maxillary left lateral incisor and left canine, consistent with the patient’s initial esthetic goals. (**a**) Full-face smile; (**b**) frontal smile view; (**c**) left lateral smile view; (**d**) intraoral frontal view; and (**e**) intraoral left lateral view demonstrating complete closure of the interproximal space.

The patient was pleased with the shade and shape of both partial ceramic veneer restorations (Figure 9).

She was provided with an occlusal guard to wear at night to protect the restorations. Oral hygiene instructions were given, and she was advised to attend annual follow-up appointments to assess the periodontal tissues and ceramic restorations. At the two-year follow-up, the patient remained satisfied with the outcome.

## 3. Results

### 3.1. Results of the Literature Review

To provide clinically oriented guidance on this emerging approach, the literature review was intentionally limited to case reports, as these publications most often provide the detailed, step-by-step clinical information needed for practical application. The selected manuscripts included only reports that clearly described the clinical protocols and outcomes of partial ceramic veneers in the anterior esthetic zone (Table 2).

Collectively, these reports highlight key elements of treatment, including diagnostic planning, patient- and defect-specific considerations (e.g., tooth alignment, diastema size, shade requirements, and occlusal factors), restorative design, bonding/cementation procedures, and finishing/polishing. They also report follow-up outcomes, such as esthetic integration, functional performance, patient satisfaction, and any complications when applicable. Overall, this evidence offers valuable insight into the real-world application, benefits, and limitations of partial ceramic veneers in the smile zone.

### 3.2. Methodological Assessment of Included Case Reports

The methodological characteristics and outcome reporting of the included case reports were analyzed to identify common procedural elements and reporting patterns. All studies described the use of partial ceramic veneers and reported a standardized clinical protocol (Table 3). Follow-up was documented in four reports, ranging from 12 to 30 months. One study involved implant therapy as part of a multidisciplinary treatment approach, and none reported completed endodontic treatment as part of the rehabilitation process.

**Table 3:** Methodological characteristics and outcome reporting of case reports on partial ceramic veneers.

Study	Study Design	Partial Veneer	Endodontic Treatment	Implant	Standardized Protocol	Follow-Up Reported	Outcome Data Available	Overall Assessment
Ceinos et al., 2018 [17]	Case report	+	–	–	+	+	+	++++
Caetano et al., 2023 [12]	Case report	+	–	–	+	+	+	++++
Fonseca et al., 2024 [18]	Case report	+	–	+	+	+	+	++++
Farias-Neto et al., 2015 [19]	Case report	+	–	–	+	–	+	+++
Duran Ojeda et al., 2024 [20]	Case report	+	–	–	+	+	+	++++

### 3.3. Results of the Case Illustration

A thoughtful clinical assessment is essential whenever a patient requires treatment in the smile zone, because even minor imperfections are easily noticed by the patient. The workflow begins with comprehensive diagnostic records, including dental photography and an intraoral scan. These baseline records allow the clinician to evaluate the initial condition and discuss the areas that need to be addressed with the patient. Digital tools can then be used to create a digital wax-up, which can be presented to the patient as a proposed outcome.

A minimally invasive preparation was performed, limited to smoothing sharp areas and rounding line angles to facilitate placement of the partial veneer. A final digital impression (intraoral scan) was then obtained to capture the preparation. The definitive partial veneers were handcrafted to achieve high esthetic outcomes and ultra-thin restorations.

**Figure 10:**
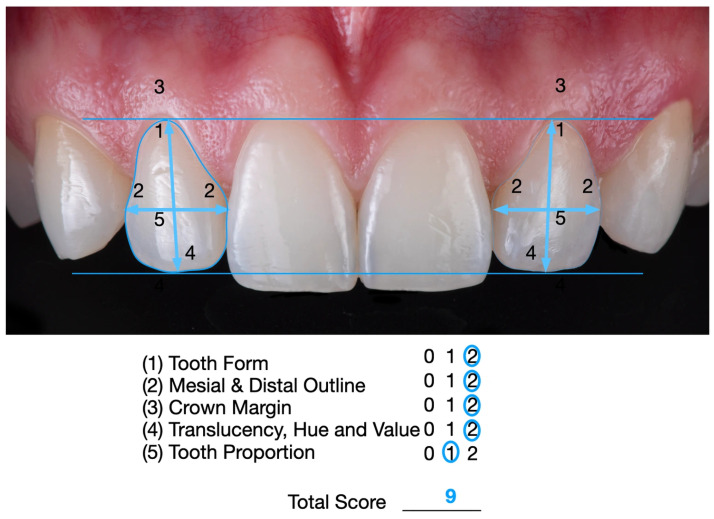
White Esthetic Score (WES) score system used to evaluate the outcome of the case illustration. The lines and arrows allow the size and shape comparison.

The White Esthetic Score (WES) is a dental index used to objectively evaluate the esthetic quality of restorations in the anterior maxilla by comparing them with adjacent natural teeth. It assesses five parameters: tooth form; mesial/distal outline; crown margin; translucency (incisal third) and hue/value (middle third); and tooth proportion. Each parameter is scored as 0, 1, or 2 (2 = ideal, 1 = acceptable, and 0 = poor) [14,15]. A total WES of 9–10 indicates a near-perfect match to the natural tooth, whereas lower scores indicate noticeable discrepancies. In the present case, the restoration achieved a WES of 9, which represents an excellent outcome (Figure 10).

The WES assessment of this case report was conducted by two prosthodontists (specialists in complex restorative dentistry) with more than 30 years of combined experience, including experience evaluating clinical outcomes using the WES system. The two evaluators performed the assessment independently and were blinded to each other’s scores; both assigned a WES of 9.

A score of 10 was not achieved because the restoration of the left maxillary lateral incisor is slightly wider than that of the contralateral lateral incisor. This modification was necessary to close the space between the left lateral incisor and the left canine. Although the left canine is also slightly wider than the right canine, this difference is not readily noticeable due to the arch form, as the distal surface is not fully visible in a frontal view. A WES of 9 indicates a very close, nearly indistinguishable match to the natural dentition and is consistent with the patient’s complete satisfaction at the end of treatment.

To support the long-term success of the treatment, the patient was instructed on an oral hygiene protocol, including proper brushing and flossing techniques with emphasis on cleaning around the restorations. The patient was also placed on a six-month maintenance schedule. Follow-up visits included dental prophylaxis and a comprehensive assessment of the restorations, the surrounding soft tissues, and overall oral health.

## 4. Discussion

The present clinical report illustrates the use of minimally invasive partial ceramic veneers to close a diastema between the maxillary left canine and lateral incisor, meeting the patient’s esthetic expectations while prioritizing tissue preservation. The goal was not merely to “fill” the space, but to achieve an undetectable integration of tooth form, gingival framing, and smile symmetry. A preliminary gingivectomy helped establish a stable gingival architecture by correcting soft-tissue contours and optimizing the gingival scaffold for the definitive restorations. The veneers were shade-matched to the adjacent dentition (value, chroma, and translucency), surface texture and characterization were refined to mimic enamel, and careful finishing/polishing produced a smooth tooth–ceramic transition to reduce plaque retention and minimize marginal detectability. Minimally invasive preparations are strongly recommended in esthetic dentistry because enamel is the most predictable substrate for durable adhesive bonding. Enamel’s favorable response to acid etching supports stable micromechanical retention, whereas dentin bonding is more technique-sensitive due to its tubular structure and higher water content, increasing the risk of incomplete resin infiltration, nanoleakage, and long-term hydrolytic degradation [21,22,23]. These challenges are particularly relevant in the esthetic zone, where marginal discrepancies or interface discoloration may compromise patient satisfaction. Conservative preparations also provide long-term flexibility by preserving tooth structure for future repair or modification (e.g., marginal staining, minor trauma, evolving esthetic demands), and they may reduce postoperative sensitivity by limiting dentin exposure [24,25,26]. In the present case, preparation was intentionally restricted to the middle third of the canine and lateral incisor, consistent with a localized, enamel-preserving philosophy. Rounded surfaces supported seating accuracy, reduced stress concentration within thin ceramics, and helped minimize the risk of chipping or crack propagation over time. Importantly, the preparation remained entirely within enamel, supporting predictable bonding and marginal stability.

At present, the literature offers limited and inconsistent guidance on indications, selection criteria, and standardized protocols for partial ceramic veneers in the esthetic zone. Unlike conventional full-facial veneers, partial designs frequently lack a definitive finish line and vary with the defect being corrected. However, available clinical reports suggest that partial veneers can be a viable conservative option for appropriately selected cases, including localized incisal wear, limited discoloration correction, contour modifications, and closure of small diastemas [27,28,29,30,31]. When executed with strict adhesive protocols, enamel-based designs can yield excellent esthetic outcomes, although success remains dependent on defect characteristics, smile line/gingival display, occlusal dynamics (including parafunction), and the clinician’s ability to achieve a smooth, biologically compatible margin. Table 1 summarizes the relevant clinical reports and applications, along with outcomes and follow-up periods [12,17,18,19]. Larger prospective studies and long-term comparative trials are still needed to define clearer evidence-based protocols. Recent evidence supports the longevity of minimally invasive veneers in selected cases. A retrospective study comparing conventional versus non-prep/minimally invasive veneers over a mean of nine years reported survival rates of 96.7% and 100%, respectively, suggesting that reduced or eliminated preparation may support favorable outcomes when case selection is appropriate [32]. Nevertheless, non-prep approaches are not universally indicated; cases requiring major color change, significant alignment correction, or substantial contour modification may still require conventional preparation to avoid overcontouring and maintain periodontal health. A prospective observational cohort study of ultrathin lithium disilicate veneers (0.1–0.2 mm) bonded to non-prepared teeth reported a 100% success rate at one year, further supporting the feasibility of ultrathin ceramics under controlled clinical conditions [33]. In the present report, the partial veneers were likewise designed to be ultra-thin (0.2–0.3 mm) to balance optical integration and contour development for diastema closure while maintaining enamel-based bonding and minimal reduction. Feldspathic ceramic was selected due to its excellent translucency and enamel-like optical behavior, which can be advantageous in thin anterior restorations where subtle characterization is required. Because feldspathic ceramic is relatively brittle, clinical performance depends on adhesive bonding, appropriate thickness distribution, adequate tooth support, and careful occlusal management—conditions best achieved with enamel-preserving designs. Retrospective data on 170 feldspathic veneers in the anterior region reported a 91.77% survival rate up to seven years, with favorable outcomes for color match, translucency, marginal adaptation, and mucosal compatibility [34]. In addition, a systematic review and meta-analysis reported a mean survival rate of 96.13% at 10.4 years across 29 studies, supporting feldspathic laminate veneers as a reliable esthetic option when properly indicated and executed [35]. Clinicians should recognize that successful partial veneers depend on careful case selection. Ideally, the teeth should be well aligned within the arch. The space to be closed should be modest; if the diastema is excessive, the restoration may appear overcontoured or unnatural. In addition, ultrathin veneers have limited ability to mask the underlying tooth shade [36,37]; therefore, if the patient desires an overall shade improvement, tooth whitening should be completed before the restorations are fabricated. Occlusion should also be carefully assessed, as heavy occlusal forces and parafunctional habits (e.g., bruxism) may increase the risk of restoration fracture [13,38].

The White Esthetic Score (WES) is a validated, reproducible index for assessing the esthetic outcome of single-tooth, tooth-supported restorations, particularly in the anterior region. The WES evaluates five domains crown form, volume/contour, color (hue and value), surface texture, and translucency by comparing the restoration with the contralateral natural tooth as the reference [39,40]. It has been widely used in the literature as an outcome measure and has demonstrated reliable performance. Notably, studies have reported good inter-observer agreement among evaluators from different clinical backgrounds, supporting the WES as a robust tool for overall esthetic assessment [41,42]. It is also important to acknowledge the limitations of the WES system, including subjectivity and limited inter-observer reliability, as results may be affected by inter-observer variability. In addition, the WES depends on the contralateral/adjacent tooth because the system relies on comparison with the natural tooth or restoration next to the treated site. If that reference tooth is not in good condition, the WES evaluation may be invalid or less reliable. Another limitation is the restricted scope of the system, as it does not assess the surrounding soft tissues, which often need to be in good condition to achieve overall high esthetic outcomes. Finally, the evaluation may be influenced by image quality, since photographs must be high quality; otherwise, they may not accurately represent the in vivo situation. In the present case report, the WES assessment was performed by two experienced dental specialists. The adjacent teeth and soft tissues were in acceptable condition, and the photographs were taken by experienced clinicians using high-quality equipment. These measures help minimize the limitations associated with the WES system.

Clinicians should also be aware that failures of ceramic veneers have been reported in the literature and should develop a treatment plan aimed at minimizing and, when possible, preventing these complications. A recent systematic review evaluated the survival of partial laminate veneers and categorized the covariates affecting their longevity. The review assessed 56 studies and reported an overall success rate of 90.81%, regardless of the follow-up interval. The most common reasons for failure were debonding, fracture, abrasion of the porcelain, staining at the tooth–porcelain margin, and secondary caries. The authors concluded that laminate veneers demonstrate high survival rates; however, outcomes may be influenced by several prognostic variables [43]. Clinicians can reduce factors that negatively affect survival through appropriate preventive strategies. For example, performing adhesive procedures under rubber dam isolation helps prevent contamination and may reduce the risk of debonding. Providing a night guard may help prevent chipping or fracture of the restorations, and regular check-ups with professional cleanings may reduce the risk of secondary caries [44,45].

As an alternative to partial ceramic veneers, partial resin composite veneers may be considered. However, the literature has reported limitations of resin composite veneers, including staining and loss of gloss, whereas dental ceramics generally demonstrate superior mechanical properties [46,47,48]. In the present report, the patient had previously received a resin composite restoration and was dissatisfied with the outcome; therefore, she requested ceramic restorations.

Minimal or partial preparation designs offer several favorable biomechanical advantages when case selection is appropriate. By preserving enamel, these restorations maximize the quality and predictability of adhesive bonding, which is strongly associated with improved resistance to debonding and marginal breakdown under functional loading. Maintaining native tooth structure helps retain tooth stiffness and reduces the extent of structural weakening that can occur with more aggressive preparations, thereby promoting more favorable stress distribution within the tooth–restoration complex [49,50]. Moreover, a recent retrospective study evaluating ultra-thin no-prep veneers without a defined margin assessed a total of 201 veneers with a 24-month follow-up. The results indicated a 99.5% survival rate, and the authors concluded that these findings support the use of minimally invasive options in anterior restorative dentistry [51].

Clinicians should also be aware of the learning curve associated with adopting new techniques. Partial-veneer workflows often require a different approach than traditional preparations, and predictable outcomes depend on a thoughtful treatment plan and a well-defined clinical sequence.

Overall, this case and the available literature support partial ceramic veneers as a conservative treatment option for appropriately selected patients. Optimized gingival architecture, enamel-preserving preparation, appropriate material selection, and meticulous finishing/polishing are key to achieving an imperceptible tooth–ceramic transition and supporting both immediate esthetic satisfaction and long-term clinical stability.

Despite the favorable esthetic outcome observed in this case, clinicians should be aware of several potential limitations associated with ultrathin partial ceramic veneers. The absence of a well-defined finish line may increase the difficulty of margin identification and complicate both laboratory fabrication and clinical finishing procedures. In addition, ultrathin ceramic restorations may be more susceptible to mechanical complications under unfavorable occlusal conditions, particularly in patients presenting parafunctional habits such as bruxism, which has been associated with increased risk of restoration fracture or failure [13,38]. Although laminate veneers generally demonstrate high survival rates, their longevity may still be influenced by multiple prognostic factors, including occlusal loading, adhesive procedures, and case selection [43]. In certain clinical situations, conservative direct composite restorations may represent a viable alternative, particularly when minor morphological corrections or diastema closure are required. However, the literature reports that resin composite veneers may present limitations such as increased susceptibility to staining and loss of surface gloss over time [46,47]. In contrast, ceramic materials tend to provide superior optical stability and favorable long-term clinical performance, which may justify their selection in carefully planned esthetic rehabilitations [34,35,44]. A key limitation of this clinical illustration is the relatively short follow-up period of two years; therefore, longer-term outcomes should be reported in future studies. In addition, this case report does not include an intra-patient comparison (e.g., a conventional full-veneer restoration placed in the same patient). When feasible, future clinical investigations should incorporate within-patient comparisons of different restorative approaches to allow a more direct and comprehensive evaluation of esthetic outcomes. Finally, because this case was performed by experienced clinicians, less experienced operators should be mindful of the learning curve associated with new techniques. Ongoing training and skill development are recommended to support safe and predictable implementation.

A further limitation relates to the review design. As a narrative review, this manuscript does not provide the highest level of evidence on the topic. Nevertheless, narrative reviews can offer important value through expert-led synthesis and critical interpretation of the literature. Although they are less methodologically prescriptive than systematic reviews, narrative reviews can integrate diverse findings, provide clinical context and perspective, clarify key concepts, and identify knowledge gaps and priorities for future research in both emerging and established fields.

## 5. Conclusions

Partial ceramic veneers in the esthetic zone represent a novel, conservative approach that differs from conventional veneer preparation designs. Careful case selection is critical and requires several factors, including teeth that are ideally positioned within the arch and a diastema that is not excessively wide, as larger spaces may require overcontouring and can make the restoration appear unnatural.

The literature review indicates that only a limited number of clinical reports are currently available; however, a thorough clinical evaluation and a well-designed treatment plan can still lead to predictable outcomes. The present case illustration details the clinical steps, including a diagnostic wax-up that allows patients to visualize and evaluate the proposed outcome before any irreversible treatment. In addition, the hand-crafted restorations achieved highly esthetic results and fulfilled the patient’s functional and esthetic expectations.

Nevertheless, the findings presented in this report should be interpreted with caution, as the level of evidence provided by a single clinical case is inherently limited. Further prospective clinical studies with larger patient samples and long-term follow-up are necessary to better define the indications, performance, and predictability of partial ceramic veneers.

## Figures and Tables

**Table 1:** Inclusion and exclusion criteria of the search performed.

Criterion	Inclusion	Exclusion
Time period	Publications available between January 2010 and December 2025	All publications published before January 2010
Language	English	Non-English.
Type of articles	All research types including case studies (e.g., case reports, case series, clinical techniques) and literature reviews (e.g., systematic reviews and meta-analysis). Full text available.	Letters, books, book chapters, and full text not available

**Table 2:** This table summarizes case reports of partial ceramic veneers in the esthetic zone [17,12,18,19].

Title, Authors and Year of Publication	Methodology	Conclusions
Esthetic rehabilitation of the smile with partial laminate veneers in an older adult. Ceinos et al. 2018 [17].	Partial lithium disilicate veneers used to treat fractured maxillary central incisors, the restorations covered the middle and incisal third of the teeth. A 1-year follow-up patient was still satisfied with the outcome.	Both teeth were restored with a natural appearance. The shape of the restorations corresponded to the patient’s esthetic expectations while ensuring a balance between the incisive edge and lower lip between the dry and moist parts to allow for good allocution and phonemes.
Partial Ceramic Veneer Technique for Challenging Esthetic Frontal Restorative Procedures. Caetano et al., 2023 [12].	A partial lithium disilicate veneer was used to address staining in the gingival and middle thirds of the maxillary left central incisor. Prior to the restoration, the patient underwent home bleaching and a gingivectomy. A 30-month follow-up showed clinically acceptable results.	A cervical partial ceramic veneer is an innovative, viable and safe approach after a short-term follow-up.
Management of Compromised Spacing in the Esthetic Zone by Combining an Ultra-Thin Partial Ceramic Veneer and a Ceramic Implant Crown: A Case Report of a Multidisciplinary Approach and Technique Description. Fonseca et al. 2024 [18].	The treatment included a partial lithium disilicate veneer covering the mesial and incisal surfaces of the maxillary right central incisor. The patient also received a dental implant with bone grafting at the site of the left central incisor.	By using a partial ceramic veneer, the spacing issue was resolved, achieving a favorable and natural match with the remaining dentition through a minimally invasive approach.
Esthetic Rehabilitation of the Smile with No-Prep Porcelain Laminates and Partial Veneers. Farias-Neto et al. 2015 [19].	The treatment included feldspathic porcelain partial veneers on both central incisors to cover the mesial surfaces and close the diastema, as well as on both maxillary canines to address incisal wear. No follow-up was provided.	No-prep veneers and ceramic fragments are an excellent rehabilitative option for situations in which the dental elements are healthy and can be modified exclusively by adding material and the patient does not want to suffer any wear on the teeth.
Clinical report and fractographic analysis of a fractured partial laminate veneer. Duran Ojeda et al. 2024 [20].	A partial lithium disilicate veneer was placed to restore the incisal, facial, and mesial surfaces of the maxillary right central incisor, in conjunction with a full crown on the left central incisor. After 18 months of service, the partial veneer fractured when the patient bit on a chicken bone.	Ceramic partial laminate veneers represent a suitable option for restoring minimal abnormalities. However, occlusal function and parafunctional habits require a comprehensive evaluation.

## Data Availability

Data presented in this study are available on request from the corresponding authors.
